# The impact of industrial synergistic agglomeration on residents’ health levels

**DOI:** 10.3389/fpubh.2024.1410359

**Published:** 2025-01-03

**Authors:** Di Qi, Wenhan Xu

**Affiliations:** ^1^School of Finance, Nanjing Agricultural University, Nanjing, China; ^2^School of Accounting, Nanjing Audit University, Nanjing, China

**Keywords:** industrial synergistic agglomeration, residents’ health levels, spatial spillover effects, green industry, traditional industry, GS2SLS method

## Abstract

**Introduction:**

This study investigates the impact of industrial synergistic agglomeration on residents’ health levels in China. It explores how green and traditional industry agglomeration models influence residents’ health levels outcomes and identifies the underlying mechanisms driving these effects.

**Methods:**

Using panel data from 283 prefecture-level cities and above in China from 2003 to 2020, the study applies the Generalized Spatial Two-Stage Least Squares (GS2SLS) method. This approach allows for a systematic analysis of both direct and spatial spillover effects, focusing on the comparative impacts of green and traditional industrial agglomeration models.

**Results:**

(i) Spatial Effects: Residents’ health levels exhibits a significant positive spatial effect, with public health improvements in one city positively influencing neighboring cities.

(ii) Industrial Agglomeration: Industrial synergistic agglomeration has a stronger positive impact on residents’ health levels compared to single-industry agglomeration.

(iii) Mechanisms: The effect of industrial synergistic agglomeration on residents’ health levels operates through three primary mechanisms:

- Population Agglomeration Effects: Enhanced population clustering contributes to better public health services.

- Media Dissemination Effects: Improved information dissemination raises public health awareness.

- Income Growth Effects: Increased income levels drive better access to healthcare and lifestyle improvements.

(iv) Model Comparison: Green industry synergistic agglomeration proves more beneficial for residents’ health levels improvement than traditional industry agglomeration.

**Discussion:**

The findings highlight the critical role of industrial synergistic agglomeration, particularly in green industries, in promoting residents’ health levels. Policymakers are encouraged to prioritize strategies fostering green industry clustering and to leverage the identified mechanisms to amplify public health benefits across regions.

## 1 Introduction

Since the reform and opening up, China’s economy has sustained rapid growth through the effective spatial concentration of economic activities. The advent of a new era has been marked by the deepening of major regional strategies such as the coordinated development of the Beijing-Tianjin-Hebei region, the development of the Yangtze River Economic Belt, the construction of the Guangdong-Hong Kong-Macao Greater Bay Area, and the ecological protection and high-quality development of the Yellow River Basin, further reinforcing the trend of spatial concentration of economic activities (1, 44). This process has not only propelled the country to achieve numerous historic accomplishments in its socio-economic sphere. In the quest for economic development, an excessive focus on economic growth has overshadowed the value of environmental resources, at times sacrificing the environment to spur economic growth. The acceleration of industrialization and urbanization has led to concentrated energy consumption, exacerbating urban air pollution, evidenced by severe declines in atmospheric visibility and frequent smog-engulfed cities (2, 3). This has manifested in public health domains, where diseases related to environmental pollution have become major “killers” affecting the health of Chinese residents, particularly noticeable in regions with high industrial concentration (4–6).

Taking the Pearl River Delta, China’s largest industrial production area, as an example, it hosts the most prosperous urban agglomerations but also suffers from severe air pollution compared to other regions in China, garnering significant concern. According to the latest statistical data, in 2023, the regional average concentration of PM2.5 in the Pearl River Delta was 45 μg/m^3^, with the average concentration in cities ranging between 35—50 μg/m^3^. With the acceleration of “regional economic integration,” the distance between regions continues to diminish, coupled with the natural attributes of long-distance diffusion of air pollution, leading to a trend of “regional pollution integration” across all cities within the region. Studies indicate that the air quality of cities in the Pearl River Delta is influenced by pollution sources from other cities within the region by approximately 10%—30%. This suggests that the air quality in each city is affected not only by local pollution sources but also by those in surrounding cities to varying degrees. Therefore, addressing environmental health issues is not merely a problem of individual administrative regions but also a regional issue that must consider the impact between cities within industrial agglomeration areas, indicating spatial dependence of air pollution problems with surrounding areas (7, 8).

The development of public health affairs is undeniably crucial for the nation. The state continuously introduces relevant public health policies, gradually elevating them to a national strategy, guiding the direction for high-quality development of public health affairs. Cities, as the core carriers of industrial synergistic agglomeration development and the construction of a healthy China, while promoting urban prosperity, can also directly or indirectly affect public health through various channels such as wealth creation, technological progress, resource supply, population movement, and environmental pollution. Therefore, elucidating how to enhance public health within industrial synergistic agglomeration at the urban scale has emerged as an urgent and significant issue.

Industrial synergistic agglomeration, an economic concept, refers to the concentrated distribution state formed within a specific geographical area, where different industries or enterprises associated with each other share resources, technology, and information to maximize production efficiency. Such agglomeration promotes synergistic development among industries, strengthens the complementarity of industrial chains, and enhances innovation and competitiveness. The emergence of industrial synergistic agglomeration, often accompanied by the dissemination of knowledge, technological innovation, and the concentration of professional talents, plays a significant role in regional economic development. This paper delves into the macro perspective of industrial synergistic agglomeration, focusing on its impact and mechanism on urban public health, thereby deriving policy implications for improving urban public health. This work holds substantial theoretical and practical value in deepening the understanding of public health evolution in line with industrial synergistic agglomeration and enhancing the public health governance capacity of the entire society. The marginal contribution of this thesis lies in systematically expounding the theoretical mechanisms by which industrial synergistic agglomeration affects urban public health and employing the Generalized Spatial Two-Stage Least Squares (GS2SLS) method to conduct a multidimensional empirical examination, taking into account the treatment of endogeneity and spatial association.

## 2 Literature review

The existing body of literature related to the research topic at hand can be methodically categorized into three primary facets. First, the negative aspects of industrial agglomeration. As the scale of industrial agglomeration continues to expand beyond the composite carrying capacity of resources, environment, and public goods provision, a series of diseconomies of agglomeration emerge. (45) employs the analytic method from regional and industry perspectives to assess the environmental health loss effects. From a regional standpoint, it evaluates the exposure levels to various pollutants in a single area and the reduction in population health loss due to energy structure optimization, as well as forecasts the future emissions of gases like SO_2_ under different energy consumption scenarios and their induced public health economic loss assessment (9). In recent years, a few scholars have conducted studies on the causal identification between these factors, with some focusing on a single region and employing the Grossman model to analyze the impact of air pollutants and other factors on residents’ health demand (10–12). Others have used panel data and econometric models to study the impact of air quality and other factors on public health (13), comparing differences in health effects between genders and between residents of developed and developing countries (14, 15). Research on 112 key cities in China reveals that the more economically backward a region is, the more severe the health economic burden of pollution is, exhibiting a clear regressive distribution (16).

Second, the consequences of compromised public health primarily explore individual income and education levels, family harmony and sanitary environments, or socio-economic factors like air pollution, social status, urbanization, and healthcare resource allocation. Furthermore, related studies often employ a single indicator related to residents’ physiological health, such as the number of deaths from respiratory diseases, to measure public health. These studies predominantly develop from three angles. Firstly, they analyze the relationship between different pollutants and health statuses, where numerous scholars have demonstrated a statistically significant and negatively correlated relationship between various pollutants and life expectancy, indicating the adverse effects of increasing pollutants on expected lifespan and diseases (11, 17). Secondly, many scholars focus on the extent of economic losses caused by health hazards. Research from different periods unanimously shows that the health economic losses caused by diseases triggered by air pollution or resulting in death are substantial (14). This is primarily based on the analytic method that evaluates the economic losses to residents’ health due to environmental pollution, considering pollutant dose effects and the population exposure-response principle (9, 18, 19). The research content mainly includes the medical and healthcare costs of diseases, the productivity loss due to premature death, and the decline in health quality (16). To mitigate environmental health losses, some scholars analyze the changes in people’s transportation behavior from perspectives like traffic, addressing the issues of environmental improvement and health cost benefits (5, 20). Thirdly, the regional imbalance of environmental pollution health impacts. When studying the impact of the environment on health, the influence of structural effects, i.e., the differential impacts of environmental pollution on the health of various regions or groups, cannot be overlooked. Research has found that the monetized disease burden generated by air pollution in developing countries is more pronounced in impoverished families (41). Some scholars have observed that pollutants affect countries at different stages of development differently and that besides pollution, socio-economic factors exert a stronger influence (21). Thus, it can be posited that the heterogeneity in environmental health performance, seemingly revolving around individual differences, is largely attributed to elements such as economic development levels and healthcare expenditures (3).

Third, the Public Health Effects of Industrial Agglomeration. The impact of industrial agglomeration on public health is a multidimensional and complex issue that encompasses economic growth, environmental changes, and social structures. From an academic standpoint, this topic can be explored through several theoretical frameworks, particularly incorporating the viewpoints of Western economists. Alfred Marshall’s theory of externalities was among the early discussions on the economic effects of industrial agglomeration, suggesting that agglomeration could bring about positive externalities, such as efficiency gains through specialization and technological spillovers. However, from a public health perspective, industrial agglomeration might also generate negative externalities, such as environmental pollution and excessive resource exploitation, which could adversely affect residents’ health. Paul Krugman’s New Economic Geography discussed the formation of industrial agglomeration and its impact on the distribution of economic activities. His model highlighted that industrial agglomeration could promote economic growth through market size effects and reduced transportation costs. Yet, he acknowledged that such agglomeration might lead to uneven resource distribution and increased environmental stress, thereby affecting public health. For instance, excessive agglomeration could exacerbate urban pollution, raising the risk of respiratory diseases among residents. Michael Porter’s theory of competitive advantage, through the “Diamond Model,” discussed the role of industrial agglomeration in fostering innovation and competitiveness. From a public health perspective, Porter’s theory implies that industrial agglomeration, by promoting innovation, could indirectly improve the services and products of the health industry, such as through advances in medical technology enhancing public health standards. However, industrial agglomeration could also result in the workforce being overly concentrated in high-intensity jobs, exacerbating occupational diseases and mental health issues.

From the perspective of environmental economics, environmental economists focus on the impact of economic activities on the environment and the effects of environmental changes on human welfare. In the context of industrial agglomeration, environmental economics pays special attention to issues such as pollution emissions, resource consumption, and ecological damage. For example, the emission of pollutants can increase the risk of disease among residents, reducing the quality of life. Environmental economics provides theories and tools for assessing and managing the environmental impacts of industrial synergistic agglomeration, aiming to minimize its negative effects on public health (10). Research by (40) indicates that single-industry agglomeration often leads to over-reliance on single resources and concentrated environmental stress, such as pollutant emissions and resource consumption, posing threats to public health. Industrial diversity and synergistic effects can promote more sustainable environmental management practices, reducing environmental pollution through technological innovation and more efficient resource use.

In summary, the impact of industrial agglomeration on public health is a multifaceted issue. The perspectives and theories of Western economists provide a framework for understanding this complex phenomenon, indicating that while industrial agglomeration has the potential to indirectly improve public health through economic development and technological innovation, it also poses risks of negative health impacts due to environmental pollution and social stress. Compared to single-industry agglomeration, the existing literature suggests that industrial synergistic agglomeration has a more positive impact on urban public health, especially in terms of promoting economic stability, reducing environmental stress, improving quality of life, and enhancing health services. Therefore, policymakers need to find a balance between promoting the economic benefits of industrial agglomeration and managing its potential negative impacts on the environment and public health.

## 3 Theoretical analysis and research hypotheses

Spatial economics examines the distribution of economic activities in geographical spaces, the factors influencing them, and their impact on the economy, emphasizing the significance of geographic location, spatial distance, and inter-regional interactions. Industrial synergistic agglomeration, a core concept within spatial economics, refers to the clustering phenomenon of similar or complementary industries within a specific geographical area. From the perspective of spatial economics, industrial synergistic agglomeration has various economic and social impacts.

Firstly, the economies of scale effect. Industrial synergistic agglomeration can achieve economies of scale, reducing production costs. When businesses cluster within a certain area, they can share infrastructure, service facilities, and labor markets, thereby reducing the average cost per unit of output. Furthermore, the close links in the supply chain also reduce transportation and transaction costs (22, 23).

Secondly, the market and labor force concentration effect. By aggregating a large number of businesses and labor, industrial synergistic agglomeration forms a larger local market, attracting more suppliers and service providers. This concentration facilitates market expansion and labor market diversification, providing businesses with abundant resources and talent support (8, 24).

Thirdly, the knowledge and technology spillover effect. Within industrial agglomeration areas, businesses can concentrate on their areas of expertise, achieving more efficient division of labor and cooperation through close collaboration along the industrial chain (25). Meanwhile, geographical proximity between businesses and industrial diversity promote the sharing and dissemination of knowledge and technology, conducive to innovation.

Fourthly, spatial imbalance and regional development disparities. While industrial synergistic agglomeration plays a positive role in promoting economic growth and technological advancement, it can also lead to regional development imbalances. Resources and talents may excessively flow to agglomeration areas, exacerbating economic disparities between different regions, creating a scenario where “the rich get richer, and the poor get poorer” (8, 21, 26). Thus, managing industrial synergistic agglomeration and coordinating regional development becomes a crucial task for policymakers.

Based on the aforementioned understanding, the impact of industrial synergistic agglomeration on urban public health can be elucidated through the following theoretical lenses: the Compact City Theory, Behavioral Economics Theory, and Urban Comprehensive Carrying Capacity Theory. In the early stages of industrial synergistic agglomeration, according to the Compact City Theory, dispersed medical resources around the city will shift toward the agglomeration area, generating economies of scale for public health resources, lowering marginal costs, and maximizing desired output (4). Additionally, the agglomeration area facilitates administrative management, aiding in more efficient public health monitoring, timely identification of pathogenic factors, and effective control. Moreover, agglomeration results in a concentrated population within a compact area, forming large-scale communities, enhancing the efficiency of individual public transportation vehicles, thereby controlling the total number of motor vehicles, reducing pollutant emissions, and improving urban air quality (25, 27). According to the theory of Behavioral Economics, the impact of industrial synergistic agglomeration on public health can be analyzed from aspects such as human behavioral preferences, cognitive limitations, and social interactions. Industrial synergistic agglomeration influences public health by altering behavioral patterns, impacting decision-making processes, and improving or deteriorating the living environment (5, 14, 28). Firstly, it promotes health awareness and behavior change. Areas of industrial synergistic agglomeration often accompany the spread of knowledge and increased social interaction. Within the context of green industrial synergistic agglomeration, concepts of environmental protection and health are more easily disseminated among groups, encouraging people to adopt healthier lifestyles and consumption habits. Secondly, it increases the accessibility of health services and facilities. The economic growth and population concentration brought about by industrial synergistic agglomeration can foster the development of health services and related facilities, such as more medical institutions, sports facilities, and parks, thereby improving public health levels (27). According to the Urban Comprehensive Carrying Capacity Theory, industrial synergistic agglomeration can promote urban economic growth, provide a multitude of employment opportunities, thereby improving residents’ living standards and social welfare, and positively impacting public health. Economic prosperity also means that more funds can be allocated for public health, healthcare, and other social services. Particularly, to support industrial synergistic agglomeration, cities often invest in the construction and renovation of infrastructure such as transportation, communication, water and electricity supply, as well as the enhancement of public services such as education, medical care, and leisure, all of which contribute to improving residents’ health levels (3, 29).

In the latter stages of industrial synergistic agglomeration, the concentration of population alongside declining profitability in industries may lead to psychological and physiological health issues among employees, such as anxiety and depression. Concurrently, areas of industrial synergistic agglomeration may foster some unhealthy lifestyle habits due to high work intensity and fast-paced life, such as irregular eating habits and lack of exercise, potentially increasing the likelihood of physiological diseases. As industries develop, environmental pollution issues in traditional areas of industrial synergistic agglomeration are likely to worsen, such as air and water pollution, with long-term exposure to adverse environments possibly leading to various health problems among residents (37). From a behavioral economics perspective, even if individuals recognize the potential harm of pollution to health, cognitive biases such as “status quo bias” may still prevent them from taking adequate preventive measures (13, 19). According to the theory of urban comprehensive carrying capacity, industrial synergistic agglomeration might lead to excessive consumption of local natural resources and exacerbation of environmental pollution, such as water shortages, air and water pollution, etc., directly impacting public health. The significant congregation of populations might result in urban overcrowding, cramped living spaces, and traffic congestion, increasing residents’ life stress and psychological pressure. The rapid growth of population and industries may surpass the expansion speed of urban infrastructure, leading to an increased burden on public services such as water supply, sewage disposal, and garbage treatment, negatively impacting public health (9). Although industrial synergistic agglomeration can promote economic growth, benefits often concentrate on specific groups, possibly exacerbating socio-economic inequality. Low-income groups may not be able to enjoy improved healthcare services, intensifying health inequality issues. According to the compact city theory, after reaching a certain stage of industrial synergistic agglomeration, the high density of buildings and limited green space in compact cities will lead to increased urban temperatures, exacerbating the urban heat island effect, negatively affecting public health (12, 15, 16, 30).

In sum, urban public health’s evolution pattern with the development of industrial synergistic agglomeration may exhibit stage-like nonlinear characteristics due to differences in the degree of agglomeration. Based on this, the following hypothesis is proposed:

*H1:* The impact of industrial synergistic agglomeration on urban public health is nonlinear, specifically manifesting a reverse U-shaped relationship.

Following theoretical deduction, it is understood that industrial synergistic agglomeration significantly impacts urban public health, leading to the emergence of a new scientific inquiry: through what mechanisms does this impact materialize? Drawing from the relevant literature and the aforementioned theoretical logic, this study identifies at least three mechanisms at play:

1. The Population Agglomeration Effect. Despite public health resources possessing the attributes of public goods, and the government playing a crucial role in resource provision, like other factors, they cannot entirely escape the characteristics of scarcity and profit-seeking. Under the mechanism of optimal choice, high-quality resources flow to areas with higher marginal returns. When the degree of industrial synergistic agglomeration is within a reasonable range, the combined forces of government public health investment and market supplementary resource provision help promote the matching of public health resources’ supply and demand, thereby positively impacting urban public health (22, 23).
2. The Media Dissemination Effect. The media typically caters to public interest, especially in regions where mobile internet is more developed, with numerous self-media platforms. Compared to official media, self-media has a higher level of public engagement. Industrial synergistic agglomeration leads to a rapid expansion of regional production scale, increasing pollutant emissions and deteriorating the regional environment. With media advocacy, environmental issues are more easily brought to widespread social attention, thus prompting the government to implement stricter environmental regulations, demanding businesses reduce pollutant emissions. This promotes urban ecological restoration and environmental governance (30, 31), which is of significant importance to improving public health.
3. The Income Growth Effect. Industrial synergistic agglomeration can form economies of scale, create more employment opportunities, and offer better labor remuneration, promoting regional *per capita* income growth. When residents have more disposable income, they will pay more attention to investing in their own health (3, 32). For example, purchasing health products, undergoing regular medical check-ups, participating in sports activities, and improving dietary habits. These actions collectively enhance the regional public health level. Furthermore, when regional residents’ income is higher, medical institutions are more motivated to improve the level of medical services, such as introducing more advanced medical equipment and drugs, employing more capable medical teams.

Hence, the following hypothesis is proposed:

*H2:* Industrial synergistic agglomeration can impact urban public health through population agglomeration effects, media dissemination effects, and income growth effects.

Since the Paris Climate Agreement, China has considered the dual carbon targets as a strategic task and key project, underlying the economic implications of reducing carbon emissions and other pollutants in traditional industries across regions and vigorously promoting the development of low-carbon green industries. Transitioning and reducing polluting industries to form low-carbon industry clusters raises the question: Do green industry and traditional industry agglomeration models have different impacts on urban public health? Existing literature lacks attention to this aspect. According to urban development principles, the process of industrial synergistic agglomeration should adapt to population size distribution. Inspired by related research and based on the compatibility between industrial synergistic agglomeration and population agglomeration (which is directly and closely related to urban public health), this study further classifies cities’ industrial synergistic agglomeration models into green industry synergistic agglomeration and traditional industry synergistic agglomeration.

From the perspective of agglomeration models, under the green industry synergistic agglomeration model, industrial synergistic agglomeration and population agglomeration occur in different areas, showing inconsistency in spatial layout and agglomeration scale (23, 33). Green industries, mainly relying on R&D personnel, therefore generally do not face labor supply shortages or excesses, can achieve refined division of labor and cooperation, and rational production layout, thereby obtaining efficient growth. In contrast, traditional industry synergistic agglomeration is characterized by population agglomeration lagging behind industrial synergistic agglomeration, often stemming from local governments’ preference for “GDP competition” and emphasizing scale expansion. This economy-first governance concept can cause factor allocation distortion and economic efficiency loss, possibly leading to urban resource and environmental overload, inducing unsustainable and unhealthy production and living styles. This, in turn, undermines the quality of industrial synergistic agglomeration, weakens its promotional effect on urban public health, and may even cause the promotional effect to be inferior to the inhibitory effect (20, 34).

In terms of environmental impact, green industries primarily encompass renewable energy, environmental technology, healthcare, biomedicine, information technology, and green manufacturing sectors, emphasizing sustainability and environmental friendliness (38). They adopt clean energy and environmental technologies to reduce emissions of pollutants and resource consumption. Such industrial synergistic agglomeration aids in improving air quality and reducing soil and water pollution, thereby exerting a positive impact on the health of urban residents (17). In contrast, traditional industry synergistic agglomeration, mainly comprising heavy industry and chemical sectors, is often associated with high carbon emissions and pollution, such as the release of atmospheric pollutants (PM2.5, sulfur dioxide, nitrogen oxides, etc.) and aquatic pollutants, which pose severe risks to human health, including respiratory and cardiovascular diseases.

From an economic benefit perspective, the economic performance of green industries surpasses that of traditional industries, with industrial synergistic agglomeration bringing more income growth and wealth creation to society. This can ensure the provision of urban public services and infrastructure, improve urban living environments, and effectively mitigate public health governance challenges caused by population and labor mobility (16), achieving a win-win goal for urban economic growth and public health governance. Additionally, from the viewpoint of urban comprehensive carrying capacity theory, in high-tech industry synergistic agglomeration areas, technological innovation not only promotes economic growth but also brings more environmentally friendly and energy-saving production methods, reducing environmental pollution and thus positively affecting public health.

*H3:* The green industry synergistic agglomeration model is more beneficial to improving urban public health compared to the traditional industry synergistic agglomeration model.

## 4 Empirical strategy

### 4.1 Baseline model and variable measurement

Research methods for examining the influencing factors of urban public health are diverse, with widely applied approaches including the Kaya identity, Logarithmic Mean Divisia Index (LMDI), and Stochastic Impacts by Regression on Population, Affluence, and Technology model (STIRPAT). The Kaya identity requires factor-by-factor analysis over time or across different periods, and is constrained by the need to maintain an identity relationship. Although the LMDI method allows for zero-residual decomposition and quantification of the contribution rate of various influencing factors for specific years, it cannot analyze factor elasticity, i.e., the change in urban public health resulting from a variation in a specific factor when other factors remain constant. While the traditional STIRPAT model only includes three factors—population, economic development level, and technology variables—and thus cannot fully capture the effects of various socio-economic factors on urban public health, it allows the estimation of each factor’s impact as a parameter, making it more flexible compared to the Kaya identity and LMDI method. Researchers can also extend the model according to their own research objectives. Considering the aim of this study—to investigate the influencing factors of urban public health in cities of different sizes—we will employ an extended STIRPAT model incorporating the latest data on industrial synergy and agglomeration at the urban scale for cities above the prefecture level in China. This approach aims to provide important parameters and scientific evidence for the formulation and implementation of China’s industrial agglomeration policies.

The STIRPAT model is an effective tool for evaluating the impact of external environmental factors. Based on existing literature using this model, it is crucial to carefully consider the model assumptions and data quality to ensure the robustness and interpretability of the research results. Generally, the application of the STIRPAT model in environmental impact analysis must meet the following requirements:

1. Data Requirements: The STIRPAT model requires sufficient and reliable time-series or cross-sectional data, including population size, economic development level, technology level, and environmental indicators. In this study, we control for five key aspects—human capital, industrial structure, unemployment level, openness, and environmental endowment—thereby ensuring the comprehensiveness of the data as required by the model.
2. Measurability and Representativeness of Variables: The variables selected for the study must accurately reflect the impact of each factor on the research object, and the measurement methods need to be appropriately chosen based on the actual conditions of the research subject. The measurement methods of variables in this study follow the approaches used by scholars such as Li et al. (34), Diodato et al. (25), and Wang et al. (19). These choices have undergone rigorous validation in the literature, ensuring the high measurability and representativeness of the variables.
3. Assumptions about Relationships between Variables: The STIRPAT model assumes that the explanatory variables independently and non-linearly influence the dependent variable, and that the elasticity of their coefficients is stable. Thus, users must carefully examine the relationships and stability among variables during application. In this study, we employ the Generalized Spatial Two-Stage Least Squares (GS2SLS) estimation method to handle spatial lag, spatial error, spatial autocorrelation, and spatial heterogeneity issues, thereby effectively capturing the non-linear and independent effects of explanatory variables on the dependent variable.
4. Scope of Application and Data Scale: The STIRPAT model has broad applicability and can be used in global, national, regional, or urban environmental impact studies. However, when data scale is limited (e.g., insufficient sample size or imbalanced data), the robustness and reliability of the estimation results may be compromised. This study selects a sample of 283 cities at or above the prefecture level in China from 2003 to 2020, providing a rich dataset that offers sufficient spatio-temporal observations for the model.

The spatial econometric model is set as follows:


$$\begin{array}{l} {\text{lnPhealth}}_{\text{it}}=\;C+\rho_{1}W\times {\text{lnPhealth}}_{\text{it}}+\alpha_{1}{\text{lnIndagg}}_{\text{it}}\\ +\alpha_{2}{\left({\text{lnIndagg}}_{\text{it}}\right)}^{2}+\alpha_{3}{\text{Control}}_{\text{it}}+\mu_{i}+\delta_{t}+\;\varepsilon_{\text{it}} \end{array}$$

Where i and t represent city and year, respectively; Phealth denotes the level of urban public health, Indagg represents the degree of industrial synergistic agglomeration, Control denotes control variables; C is the constant term, W represents the spatial weight matrix, ρ_1_ and α_1_-α_3_ are parameters to be estimated, μ_i_ and δ_t_ represent city fixed effects and time fixed effects, respectively, and ε_it_ is the random disturbance term. The measurement methods for variables in the model are as follows.

#### 4.1.1 Public health levels (Phealth)

This study employs the entropy-weighted TOPSIS method to measure residents’ health levels. As a multi-criteria evaluation approach, this method objectively determines indicator weights and ranks samples by comparing them to ideal solutions, thereby assessing urban public health levels. The entropy-weighted TOPSIS method effectively minimizes subjective interference, captures the multidimensional nature of health levels, and provides a scientific basis for regional comparisons and policy optimization. This paper details the core computational process and practical applications of this method, as described below:

First, an indicator system encompassing three dimensions—health outcomes, healthcare resources, and the health environment—is established. Health outcome indicators include life expectancy and the prevalence of chronic diseases. Healthcare resource indicators measure the distribution and accessibility of health resources, such as the number of physicians and hospital beds per 1,000 residents. Health environment indicators focus on external factors influencing health, such as the proportion of days with good air quality and per capita green space in urban areas.

Second, to eliminate differences in units and scales among indicators and enhance data comparability, the raw data must be standardized. The standardization method varies depending on the nature of the indicator. For positive indicators (where higher values are better, such as life expectancy), normalization involves subtracting the minimum value from the current value and dividing by the range. This transforms the data to a [0,1] scale. For negative indicators (where lower values are better, such as the prevalence of chronic diseases), normalization involves subtracting the current value from the maximum value and dividing by the range. This process unifies indicators of different units and scales, laying a consistent foundation for subsequent calculations.

Third, the entropy weighting method is used to objectively determine the weight of each indicator. This method relies on the distribution of the data itself, avoiding subjective weighting biases. First, the proportion of standardized values for each sample under a given indicator is calculated. Then, based on these proportions, the information entropy is computed. A higher entropy value indicates less variability and, thus, less significance for the indicator, resulting in a lower weight. Finally, the weights are determined inversely to the entropy values and normalized to ensure that their sum equals one. This process scientifically quantifies the contribution of each indicator to the overall health level.

After determining the weights, the next step is to construct the positive and negative ideal solutions. The positive ideal solution represents the optimal state of all indicators across all samples, defined as the maximum value for each indicator. Conversely, the negative ideal solution represents the worst state, defined as the minimum value for each indicator. These ideal solutions serve as reference points for subsequent distance calculations. By comparing the distance of each sample from the positive and negative ideal solutions, the relative performance of their health levels can be assessed

The distances between samples and the ideal solutions are calculated using the Euclidean distance formula, taking into account the weights of each indicator. The distance to the positive ideal solution measures how far a sample deviates from the optimal state, while the distance to the negative ideal solution measures how far a sample is from the worst state. A smaller distance to the positive ideal solution and a larger distance to the negative ideal solution indicate better health performance.

Finally, the relative closeness of each sample to the ideal solutions is calculated based on these distances. The relative closeness is the ratio of the negative distance to the total distance (sum of positive and negative distances), reflecting the relative quality of health levels among samples. A higher relative closeness indicates that the sample’s health level is closer to the ideal state and ranks higher. The results of this calculation can be used to rank public health levels across regions, identifying areas with superior or poorer health performance.

#### 4.1.2 Human capital (Hcap)

Tseng and Olsen (3) highlighted that human capital is regarded as a crucial factor in improving public health. Higher levels of education and human capital can enhance residents’ health awareness, strengthen their ability to manage health, and promote healthier lifestyles, thereby improving overall health outcomes. Consequently, human capital has a positive effect on public health. Cities with higher educational attainment tend to have better health outcomes, with relatively lower mortality and morbidity rates. By controlling for human capital, we eliminate the health effects driven by differences in educational levels, ensuring that the health impacts of industrial synergy and agglomeration are not obscured or distorted by variations in education. In line with existing literature, human capital is measured by the number of university students per 10,000 people.

To examine the spatial spillover effects, this study constructs a geographical distance-based spatial weight matrix (W_1_) and a nested weight matrix of geographical and economic distances (W_2_). The elements W_ij_ in W_1_ represent the reciprocal of the shortest road distance between the capitals of regions i and j. Considering that the geographical distance spatial weight matrix only reflects the impact of geographical factors on the spatial distribution characteristics of haze pollution, referring to (43), we set W_2_ = *ω*W_1_ + (1-ω) W_3_, where ω represents the weight of the geographical distance spatial weight matrix and is set to 0.5; W_3_ represents the economic distance spatial weight matrix, with its elements W_ij_ being the reciprocal of the absolute difference in the annual average *per capita* GDP between regions i and j. The weight matrix W_1_ is used in the baseline regression test, while W_2_ is used in the robustness test.

#### 4.1.3 Industrial structure (Indus)

Industrial structure (measured by the proportion of industrial value added) directly influences the environmental quality and health levels of urban residents. High degrees of industrialization are typically associated with higher levels of pollutant emissions (e.g., air and water pollution), which negatively impact public health. In contrast, regions with a higher proportion of tertiary industries (e.g., service and information technology sectors) generally exhibit better health outcomes. According to existing studies, the impact of industrial structure on public health usually manifests in a negative relationship between the degree of industrialization and health outcomes. Controlling for industrial structure helps eliminate potential confounding effects from differences in industrial composition on health outcomes. Failure to account for industrial structure could lead to misattribution of health impacts due to changes in industrial composition to the effects of industrial agglomeration. Following existing literature, industrial structure is represented by the proportion of value added by the industrial sector.

The degree of industrial synergistic agglomeration (Indagg) is measured using the entropy index in this study. This index, originating from the concept of entropy in information theory, measures industry diversity and balance. It describes the uncertainty or complexity of a system. In industrial economics, the entropy index is utilized to measure the diversity of an industry structure and the balance of its distribution within a region, indirectly reflecting the situation of industrial synergistic agglomeration. Specifically, a higher entropy index indicates a more diversified industrial structure and a more balanced distribution of industries within a region, implying a higher degree of industrial synergistic agglomeration; conversely, a lower entropy index may mean that one industry or a few industries dominate the regional economy, showing characteristics of no industry agglomeration or single industry agglomeration.

The calculation method for the entropy index is as follows:


$$E=-\sum_{i=1}^{n}p_{i}\ln\left(p_{i}\right)$$

The entropy index E is defined, where n represents the total number of industries, and P_i_ is the proportion of employment in the i industry to the total employment across all industries. The product of P_i_ and ln (P_i_) represents the relative size of an individual industry, reflecting its contribution to the overall industry diversity. Summing this across all industries yields the total contribution to industry diversity for the entire economy or region. Given that P_i_ ranges between 0 and 1, ln (P_i_) is always negative. To ensure the index is positive, the overall value is negated in this study. In empirical examinations, the entropy index is used as the measure of the degree of industrial synergistic agglomeration in the baseline regression, with employment density serving as a proxy variable for the degree of industrial synergistic agglomeration in robustness tests. This measurement approach may have the following limitations: the entropy index cannot differentiate the varying impacts of different types of industries on public health. For example, the mechanisms through which heavy industrial agglomeration and green industrial agglomeration affect public health are significantly distinct, yet the entropy index fails to directly capture this divergence. Similarly, using employment density as a proxy for industrial agglomeration is inadequate for assessing the synergistic effects of agglomeration, as it only reflects the degree of labor concentration but does not effectively capture knowledge spillovers and technological innovation effects.

In the model, the following variables and factors are controlled for specific reasons outlined below.

#### 4.1.4 Unemployment rate (Unemp)

The impact of the unemployment rate on health is complex. On the one hand, high unemployment increases residents’ economic and psychological stress, negatively affecting both mental and physical health. On the other hand, unemployment may also lead individuals to alter their lifestyles (e.g., reducing work-related stress or increasing time for exercise), potentially enhancing health in certain situations. Therefore, the effect of unemployment on public health may be bidirectional. However, in general, higher unemployment rates are usually associated with poorer health conditions. By controlling for unemployment, we ensure that fluctuations in economic conditions (particularly changes in unemployment rates) do not obscure or distort the agglomeration effect on public health, allowing for a more precise identification of the independent effects of industrial agglomeration on health outcomes. Drawing from existing research, the unemployment rate is measured by the number of registered unemployed persons in urban areas at year-end.

#### 4.1.5 Openness (Open)

Openness reflects the scale of economic activity and the mobility of the population in a city. Higher openness indicates greater international and domestic mobility, which may introduce health risks (e.g., the spread of infectious diseases). Additionally, regions with higher openness tend to have more dynamic economies, providing better access to health resources and medical technologies, thereby enhancing health outcomes. As such, the effect of openness on health is twofold: it may increase health risks on the one hand while improving access to health resources on the other. By controlling for openness, we can avoid mistaking differences in health outcomes arising from population mobility and international interactions as the effects of industrial synergy. In line with existing literature, openness is measured by travel intensity, defined as the ratio of total passenger volume to the total urban population.

#### 4.1.6 Environmental Endowment (Envir)

Environmental endowment (measured by the green coverage rate) has a direct impact on public health. High environmental quality significantly improves residents’ living conditions and health, especially in industrial cities, where good environmental quality can partially offset the adverse effects of industrial pollution. Therefore, environmental endowment generally has a positive impact on public health, and regions with higher environmental quality tend to have better health outcomes. By controlling for environmental endowment, we eliminate the influence of differences in environmental quality on health outcomes, ensuring that the health effects of industrial agglomeration are not misinterpreted due to variations in environmental quality. Consistent with the literature, environmental endowment is measured by the green coverage rate in the built-up areas.

#### 4.1.7 Spatial Lag of Public Health (W × lnPhealth)

The spatial lag captures the influence of neighboring cities’ health levels on the health outcomes of the focal city. Poor health conditions in neighboring cities may affect the health level of the focal city through mechanisms such as transboundary pollution or resource competition. Consequently, the effect of neighboring cities’ health levels on the focal city may be positive or negative, depending on the direction of the spatial spillover effect. Introducing the spatial lag term allows for better identification of intercity spatial interactions, preventing estimation bias arising from omitted spatial dependencies.

#### 4.1.8 Time dummies and city dummies

Time dummies control for macroeconomic fluctuations, policy changes, and specific yearly effects, while city dummies control for unobserved heterogeneity across cities (e.g., culture, history, local policies). Including these dummy variables helps eliminate the influence of unobserved systematic differences on health outcomes, thereby improving the robustness of the model estimates.

Each control variable affects public health through distinct mechanisms. By accounting for these variables, we can more accurately evaluate the net effects of industrial synergy and agglomeration on public health. This approach ensures that the primary effect (industrial synergy effect) is not distorted or overstated due to interference from other factors.

### 4.2 Data description and collinearity test

The raw data for the indicators involved in this study are derived from the corresponding year’s “China Health and Health Statistics Yearbook,” the EPS Data Platform, and the Center for Social and Economic Data and Applications at Columbia University. The research sample consists of 283 prefecture-level and above cities in China. To avoid potential biases caused by outliers, the raw data were processed as follows: (1) Cities with severe data deficiencies were excluded from the sample; (2) Missing data for cities with minor deficiencies were imputed using trend extrapolation and moving average methods; (3) After a comprehensive assessment of data distribution for cities with relatively complete data, the study period was set from 2003 to 2020; (4) During empirical analysis, non-ratio data were transformed using logarithmic form. Descriptive statistics of the main variables are presented in Table 1. Given the potential for multicollinearity among explanatory variables, which could affect the accuracy of model parameter estimation, the Variance Inflation Factor (VIF) is used to test this issue. Results show that all variables have VIF values less than 6, with an average VIF of 2.47, far from the threshold of 10, indicating no severe multicollinearity in the empirical model.

**Table 1:** Descriptive statistics.

Variable	Mean	S.D	Min	Max
Phealth	2. 652	2. 179	0. 156	30. 754
Indagg	3. 164	0. 559	0. 002	23. 973
Pgdp	3. 133	3. 483	0. 174	32. 671
Ecoenv	42. 121	17. 934	3. 131	110. 122
Assign	0. 917	0. 081	0. 001	0. 9,993
Hcap	1. 762	0. 538	0. 001	11. 792
Indus	47. 187	11. 345	10. 650	90. 971
Unemp	2. 916	27. 265	0. 077	1881. 213
Open	27. 125	41. 539	2. 384	649. 661
Envir	35. 366	14. 342	0. 025	386. 642

### 4.3 Rationale for choosing the GS2SLS model

In urban public health research (e.g., the spread of infectious diseases and public health investments), residents’ health conditions often exhibit significant spatial autocorrelation within the same or neighboring geographic regions. Conventional OLS regressions may result in severe estimation bias and fail to capture spatial effects adequately. Moreover, OLS models are unable to account for all surrounding environmental factors, leading to the potential endogeneity between health investments and unobserved local environmental characteristics, thereby affecting the reliability of the estimation results. To address spatial dependence and endogeneity issues, this study employs the Generalized Spatial Two-Stage Least Squares (GS2SLS) method, which allows the inclusion of instrumental variables, spatial lags, and spatial error terms in the model.

Although some scholars use Spatial Autoregressive Models (SAR) and Spatial Error Models (SEM) to account for spatial effects, these models are unable to address the estimation biases arising from endogeneity in the research context of this study. By introducing instrumental variables, the GS2SLS model can capture spatial effects while simultaneously addressing endogeneity problems. In particular, when dealing with spatial lag terms, GS2SLS is more robust compared to SAR and SEM. Additionally, the Generalized Method of Moments (GMM), widely used in the literature to address endogeneity, is unable to accommodate the complex spatial effects present in this study. Therefore, GS2SLS is the most suitable approach for the research objectives and data structure of this study.

The empirical model must consider potential spatial effects in urban public health and face the issue of potential endogeneity arising from a possible reverse causality between industrial synergistic agglomeration and urban public health. In other words, urban public health may feedback into industrial synergistic agglomeration. If urban public health management spirals out of control, it may impact the normal economic operations within the jurisdiction, leading to business failures and unemployment, capital flight, and even economically unethical regional exclusion, ultimately hindering the process of industrial synergistic agglomeration. Conversely, if urban public health issues can be effectively controlled by local governments and societal health quality continuously improves, it can demonstrate the flexibility and completeness of the urban governance system and capability, giving confidence to businesses and consumers among market entities, thereby attracting economic factors to flow in and enhance the degree of agglomeration. The presence of the aforementioned issues renders traditional parameter estimation methods ineffective, while the Generalized Spatial Two-Stage Least Squares (GS2SLS) can effectively address the challenges of simultaneous spatial effects and endogeneity (35, 36). GS2SLS uses the explanatory variables and their spatial lags as instrumental variables, estimating the spatial econometric model based on Two-Stage Least Squares (2SLS), controlling for the spatial effects of urban public health and the endogeneity arising from the reverse causality, with the obtained parameters being closer to economic reality compared to traditional methods. In estimating parameters for the empirical model, existing research is referenced, using up to the third-order spatial lag as instrumental variables for the model. Furthermore, when employing the GS2SLS estimation method, the selection of the spatial weight matrix (W) must be resolved. Considering that economic factors are increasingly breaking through geographical scales to produce spatial correlations among economic variables, the empirical process primarily utilizes the economic distance weight matrix in regression, measured by the reciprocal of the absolute difference in the annual average *per capita* regional GDP between cities during the sample period.

## 5 Empirical results

### 5.1 Baseline regression results

Table 2 presents the regression results of urban public health on industrial synergistic agglomeration based on GS2SLS. Column (1) considers only the impact of industrial synergistic agglomeration on urban public health, while columns (2) and (3) introduce other control variables. The Hausman test indicates that a fixed-effect model is appropriate. Firstly, as shown in columns (1) and (2), regardless of whether control variables are considered, the regression coefficient of lnIndagg^2^ is significantly negative, indicating an inverse “U”-shaped relationship. This confirms the nonlinear relationship between industrial synergistic agglomeration and urban public health, supporting Hypothesis 1. Since the degree of industrial synergistic agglomeration during the sample period is far below its turning point, it suggests that the industrial agglomeration in Chinese cities is still in an early stage. Therefore, the baseline regression results are based on column (3).

**Table 2:** Regression results.

Explanation variables	LnPhealth		
	(1)	(2)	(3)
W × lnPhealth	0.835***	0.832***	0.817***
	(7.54)	(7.40)	(7.82)
lnIndagg	0.231***	0.229***	0.220***
	(8.22)	(9.13)	(5.13)
lnIndagg^2^	−0.005**	−0.004***	
	(−2.16)	(−3.29)	
Turning point	19.321	22.482	
Controls	no	yes	yes
City-fixed effect	yes	yes	yes
Time-fixed effect	yes	yes	yes
R^2^	0.612	0.638	0.650
Observations	6,037	6,037	6,037

***p < 0.01, **p < 0.05, *p < 0.1.

Secondly, the coefficients of the interaction term between the spatial weight matrix and public health are significantly positive at the 1% confidence level across all columns, indicating a significant positive spatial effect of urban public health in China. This suggests that there is a same-nature mutual influence on public health between a city and its neighboring cities. This is related to natural factors such as prevailing wind directions and meteorological conditions, as well as the result of economic mechanisms such as people-to-people and economic exchanges and regional environmental protection integration. The coefficient of industrial synergistic agglomeration suggests that a 1% increase in the degree of industrial synergistic agglomeration corresponds to a 0.22% improvement in the level of urban public health. This indicates that industrial synergistic agglomeration is a crucial factor affecting urban public health and has a promotive effect, consistent with the view that industrial synergistic agglomeration in China has not been excessive and should continue to be strengthened.

**Figure 1:**
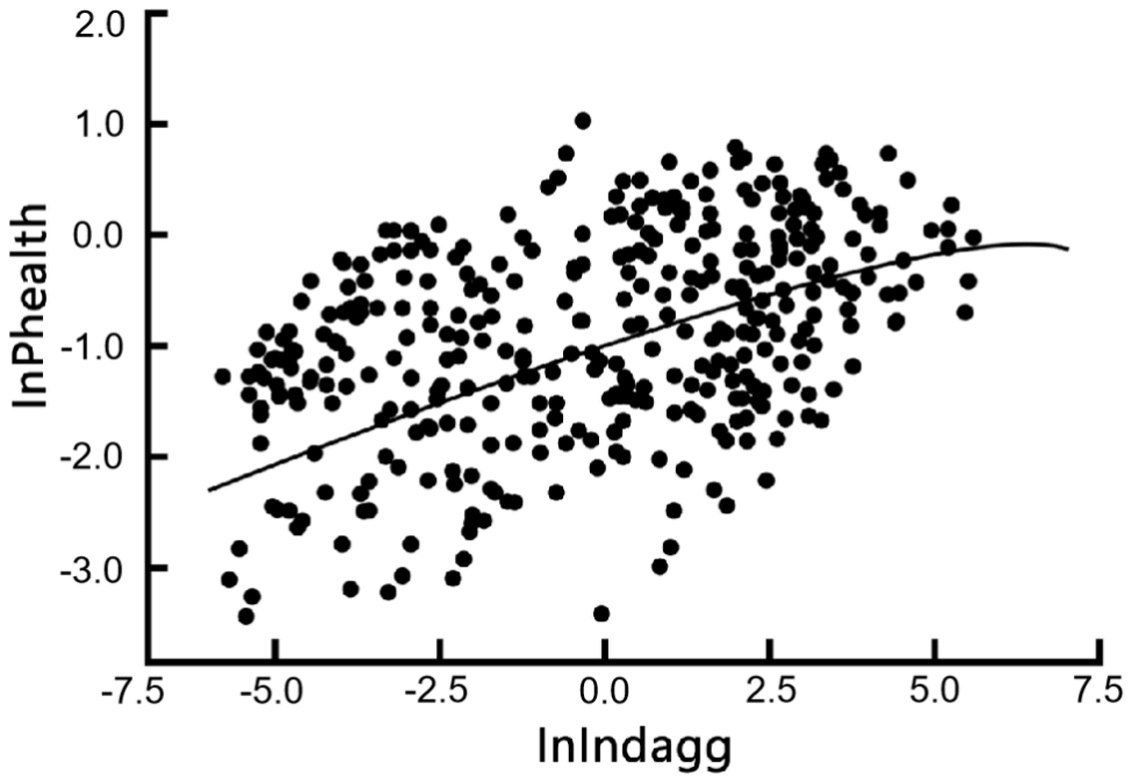
Scatter plot and fitted line of industrial synergistic agglomeration and urban public health.

Industrial synergistic agglomeration presents both challenges and contributions to urban public health. On the one hand, the agglomeration effect can intensify resource depletion and environmental pollution, increasing the ecological burden on cities. Large-scale population mobility may complicate urban social structures and exacerbate shortages in public services, creating conditions conducive to the spread of infectious diseases and healthcare resource shortages. These challenges are particularly pronounced in cities with underdeveloped health infrastructure. On the other hand, industrial synergistic agglomeration offers opportunities to enhance urban public health. Economic and social transformation driven by agglomeration fosters demand for green and healthy living environments, encouraging greater investment in health resources. Mechanisms such as income growth, ecological governance, and health education contribute to improved urban management and public health awareness. Additionally, agglomeration facilitates efficient public health services through centralized regulation and optimized resource allocation. With scientific planning and effective governance, the health-promoting effects of industrial synergistic agglomeration can be amplified while mitigating its associated risks. According to the fitting results shown in Figure 1, the public health promotion effect generated by the process of industrial synergistic agglomeration in China far exceeds its inhibitory effect, ultimately manifesting as a significant positive effect of industrial synergistic agglomeration on urban public health, thereby achieving “progress toward health through agglomeration.” However, caution is still warranted against the negative impacts on public health that may arise after industrial synergistic agglomeration reaches its turning point.

### 5.2 Robustness tests

To enhance the credibility of the baseline regression results, the article adopts adjustments to the instrumental variables, substitutes the spatial weight matrix, and replaces the core variables to re-estimate using GS2SLS, as presented in Table 3. Columns (1) and (2) use the highest second-order spatial lag terms as the model’s instrumental variables; columns (3) and (4) incorporate geographical factors into the consideration of spatial effects, namely, substituting the economic distance weight matrix mentioned earlier with a nested weight matrix of geographical and economic distances. Columns (5) and (6) use employment density as a proxy variable for industrial synergistic agglomeration; as shown in Table 3, significant spatial effects on urban public health remain evident, and the nonlinear relationship between industrial synergistic agglomeration and urban public health is validated. The impact of industrial synergistic agglomeration on urban public health continues to show a promotive characteristic over a prolonged period (currently far from reaching the turning point), demonstrating the robustness of the baseline regression results.

**Table 3:** Robustness tests.

Explanation variables	Adjusting instrumental variable		Substitution of spatial weight matrix		Substitution of explanation variables	
	(1)	(2)	(3)	(4)	(5)	(6)
W × lnPhealth	0.835***	0.802***	0.827***	0.866***	0.824***	0.903***
	(7.54)	(6.40)	(7.82)	(7.12)	(6.74)	(9.10)
lnIndagg	0.621***	0.582***	0.220***	0.577***	0.614***	0.336***
	(8.22)	(9.13)	(5.13)	(8.22)	(6.55)	(4.92)
lnIndagg^2^	−0.005**		−0.028***		−0.004***	
	(−2.16)		(−3.56)		(−3.29)	
Turning point	59.321		62.115		79.482	
Controls	no	yes	yes	no	yes	yes
City-fixed effect	yes	yes	yes	yes	yes	yes
Time-fixed effect	yes	yes	yes	yes	yes	yes
R^2^	0.612	0.638	0.650	0.633	0.649	0.610
Observations	6,037	6,037	6,037	6,037	6,037	6,037

***p < 0.01, **p < 0.05, *p < 0.1.

### 5.3 Testing hypothesis 2

To validate whether economic agglomeration can impact urban public health through mechanisms such as population agglomeration, media dissemination, and income growth, the study conducts moderation regression analysis on these variables based on the baseline regression and following mainstream mechanism testing literature. Population agglomeration (Popu) is measured by the ratio of the population in the industrial synergistic agglomeration area to the city’s total population for that year. Media dissemination (Media) is measured by the number of negative environmental reports by the media in that year. Income growth (Income) is the growth rate of income in the industrial synergistic agglomeration area compared to the same period in the previous year. Regression results are presented in Table 4.

**Table 4:** Regression results of H2.

Explanation variables	LnPhealth		
	Population	Media	Income
	(1)	(2)	(3)
W × lnPhealth	0.822***	0.823***	0.833***
	(6.47)	(6.52)	(6.50)
lnIndagg	0.681***	0.602***	0.633***
	(8.21)	(7.33)	(7.91)
lnIndagg×Popu	0.035***		
	(5.77)		
lnIndagg×Media		0.014***	
		(6.12)	
lnIndagg×Income			0.037***
			(6.05)
Controls	no	yes	yes
City-fixed effect	yes	yes	yes
Time-fixed effect	yes	yes	yes
R^2^	0.615	0.621	0.625
Observations	6,037	6,037	6,037

***p < 0.01, **p < 0.05, *p < 0.1.

Table 4, column (1) shows that the interaction term between industrial synergistic agglomeration and population agglomeration has a positive impact coefficient on public health at the 1% confidence level, indicating a significant promotive effect of population agglomeration on the relationship between industrial synergistic agglomeration and public health. Population agglomeration can provide enhanced scale effects for the healthcare sector, leading to a tilt of medical resources toward that area. When the degree of industrial synergistic agglomeration is within a reasonable range, the combined force of government public health investment and market supplementary resource provision helps to promote the matching of public health resources’ supply and demand, thereby positively affecting urban public health. This implies that the growth effect of the population is an important mechanism through which economic agglomeration promotes improvements in public health. However, this result might be attributed to the current level of industrial synergistic agglomeration in China not yet reaching the turning point that impacts public health negatively. When the pace of industrial synergistic agglomeration and population agglomeration becomes imbalanced, the inflated population will negatively affect public health.

Table 4, column (2) indicates that the interaction term between industrial synergistic agglomeration and media dissemination has a positive impact coefficient on public health at the 1% confidence level, showing a significant promotive effect of media dissemination on the relationship between industrial synergistic agglomeration and public health. This is because industrial synergistic agglomeration leads to a rapid expansion of regional production scale, increasing pollutant emissions and worsening the regional environment. According to (42), a 1% increase in the degree of industrial synergistic agglomeration can lead to a 0.1753% increase in the annual average concentration of PM 2.5, demonstrating the negative effects of industrial synergistic agglomeration on urban ecological environment governance. Media attention to this issue will make environmental problems more easily noticed by society, thereby prompting the government to issue stricter environmental regulations, demanding businesses to reduce pollutant emissions. This promotes urban ecological restoration and environmental governance, significantly improving public health.

Table 4, column (3) reveals that the interaction term between industrial synergistic agglomeration and income growth has a positive impact coefficient on public health at the 1% confidence level, showing a significant promotive effect of income growth on the relationship between industrial synergistic agglomeration and public health. This is because industrial synergistic agglomeration can form economies of scale, create more employment opportunities, and provide better labor remuneration, promoting regional *per capita* income growth. When residents have more disposable income, they will pay more attention to investing in their own health, such as purchasing health products, undergoing regular medical check-ups, participating in sports activities, and improving dietary habits. These actions collectively enhance the regional public health level. Furthermore, when regional residents’ income is higher, medical institutions are more motivated to improve the level of medical services, such as introducing more advanced medical equipment and drugs, and hiring more capable medical teams.

Thus, Research Hypothesis 2 is confirmed.

### 5.4 Testing hypothesis 3

The Difference-in-Differences (DID) model can be employed to evaluate the causal effects of policies or specific events on public health. By comparing the changes in outcomes between a “treatment group” (which is exposed to the policy or event) and a “control group” (which is not exposed), before and after the intervention, the DID model identifies the net impact of the policy or event on urban public health. This approach effectively eliminates the influence of time trends and group-specific differences, isolating the causal effect of the intervention. Existing literature has demonstrated that the DID model is a valuable tool in urban public health research, enabling robust identification of the causal impacts of policies and events on health outcomes. In the test of Hypothesis 3, this study employs the DID model to compare the effects of green industrial synergy and traditional industrial synergy on urban public health, highlighting the differences between the two modes of agglomeration.

In July 2014, China’s National Development and Reform Commission issued a notification on launching low-carbon province and city pilot projects in five provinces and eight cities to consider the policy’s impact on economic development. Over 10 years, through three revisions, the low-carbon city pilot policy gradually established the following objectives: (1) optimizing the energy structure; (2) achieving energy-saving and efficiency; (3) implementing a carbon emission target responsibility system; (4) allocating emission reduction tasks; (5) developing a low-carbon industry system; (6) controlling total carbon emissions; (7) promoting low-carbon technological innovation; and (8) nurturing emerging industries. These objectives had the following consequences: First, the development of some traditional industries would be restricted. Pilot areas would impose regulatory measures on high-energy-consuming and high-emitting enterprises, such as limiting production time and pollution emissions, severely affecting business volumes and, consequently, the entire supply chain. Second, enterprises’ pollution behavior would incur higher tax burdens. In January 2018, China formally enacted the Environmental Protection Tax Law, setting tax standards for atmospheric pollutants, water pollutants, solid waste, and noise. According to this study, the introduction of the environmental tax tripled the tax burden for enterprises on average. Since the environmental tax does not distinguish between the founding years of enterprises and lacks relief measures for startups, it increases the entrepreneurship cost. The regional government’s tax and fee reduction benefits for startups are negated by the environmental tax, significantly increasing operational costs for startups. Third, the energy-saving and emission reduction costs for traditional industry enterprises in pilot areas would significantly increase. As mentioned, the environmental tax tripled the cost of enterprise pollution, requiring large enterprises to undertake front-end and end-treatment measures (37, 39). Front-end treatment refers to the use of new energy sources to reduce the consumption of fossil fuels and, thus, atmospheric pollutant emissions; end-treatment involves decomposing all pollutants from industrial production to reduce emissions of water pollutants, solid waste, and noise. These measures necessitate the purchase of new equipment and the introduction of new technologies, substantially increasing production costs. High-polluting enterprises, generally core companies in the regional supply chain, require startups to rely on orders from these core companies for development. With significantly increased production costs for core enterprises, startups in a weaker negotiating position will see their profits substantially squeezed, inhibiting the development of the traditional manufacturing supply chain.

The central government encourages pilot cities to explore green innovation technologies, increase related research and development investments, and support the development of industries with low energy consumption and emissions. This could lead to significant differences in the characteristics of industrial synergistic agglomeration between pilot and non-pilot cities for the following reasons: Firstly, the Organisation for Economic Co-operation and Development (OECD) defines green innovation as new or improved products and innovative processes for environmental sustainability, involving technology fields such as clean energy, biodegradation, resource recycling, and pollution treatment. These technologies have distinct intellectual property characteristics, and their owners can establish new companies based on the technology to cooperate with external entities through technology transfer, patent use, consulting services, etc. Under stricter environmental regulatory pressures, governments in low-carbon pilot areas urgently need green technologies to help enterprises reduce production costs and increase business volumes, thereby ensuring stable economic growth. Thus, the government needs new enterprises specializing in green innovation to help the city’s industrial chain undergo green transformation and upgrading. In this process, the government will inevitably introduce new industrial promotion policies and increase research funding and policy support for enterprises engaged in green innovation. Secondly, the goals of the low-carbon city pilot policy can also be achieved through the construction of new urban districts. New districts can significantly reduce carbon emissions per unit area and, through more scientific planning and more precise industrial guidance, transform new areas into environmentally friendly demonstration zones, leading the city’s future low-carbon development direction. This significantly improves urban public health in the industrial synergistic agglomeration areas of pilot cities. Since this policy started in 2014, this study uses 2014 as the starting point for policy research, examining the difference in regression coefficients for the degree of industrial synergistic agglomeration on public health between the treatment group (treat) and control group (Control) three years before and after the policy. Here, the treatment group (treat) refers to cities included in the low-carbon city pilot; the control group (Control) refers to cities not included.

A Difference-in-Differences (DID) test was conducted on the matched samples, and the results are shown in Table 5. Regarding the degree of industrial synergistic agglomeration (lnIndagg), before the implementation of the low-carbon city pilot policy, the difference in coefficients between the Treat and Control groups was 0.007, with a T-value of 0.49, indicating no significant difference. After the implementation of the low-carbon city pilot policy, the coefficient difference between the Treat and Control groups reached −0.066, with a T-value of −3.13, significant at the 1% level. The Treat group’s coefficient was significantly higher than that of the Control group. Looking at the intra-group differences, the coefficient for the Control group increased significantly (from 0.531 to 0.599), while the change in innovation investment for the Control group was not significant (from 0.538 to 0.533). Overall, the difference between the two groups before and after the event was −0.066, with a T-value of 2.04, significant at the 5% level. This indicates that the implementation of the low-carbon city pilot policy enhanced the effect of industrial synergistic agglomeration on improving public health levels. Hypothesis 3 is thus confirmed.

**Table 5:** DID results of H3.

Variables	Before low-carbon policy			After low-carbon policy			DID
	Treat	Control	T-C	Treat	Control	T-C	
lnIndagg	0.531	0.538	0.007	0.599	0.533	−0.066***	−0.073***
S.D.			0.036			0.035	0.001
T-value			0.49			−3.13	−2.04
*p*-value			0.62			0.63	0.042

***p < 0.01, **p < 0.05, *p < 0.1.

**Figure 2:**
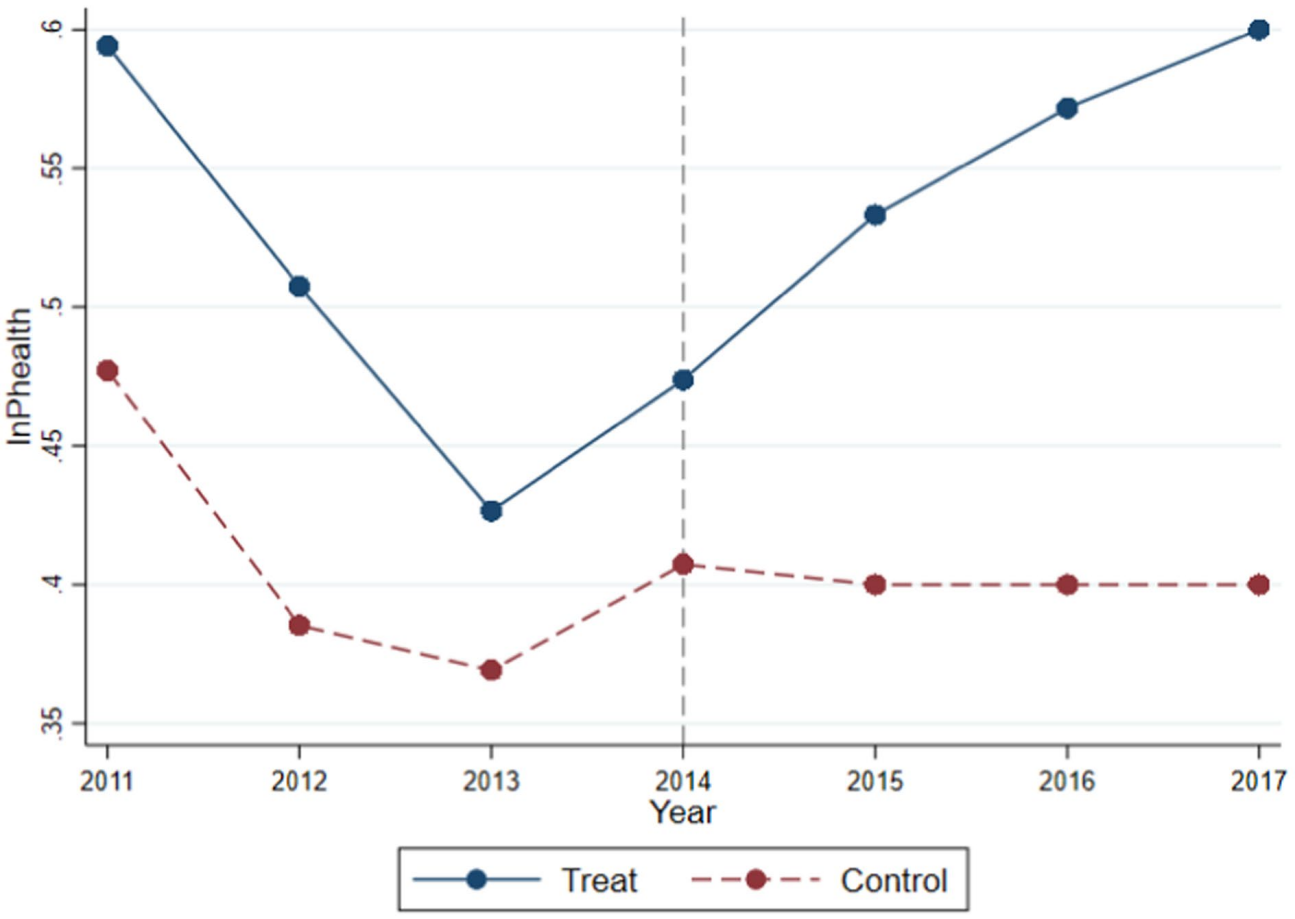
Parallel trend.

This study conducted a parallel trends analysis on the aforementioned regression results, as shown in Figure 2. It is observed that, before 2014, the coefficients of industrial synergistic agglomeration and public health levels in both the Treat and Control groups maintained a consistent trend, showing a certain downward trend over time. This may be attributed to the gradual recovery of the Chinese economy after the global economic crisis post-2010, leading to a gradual increase in the degree of industrial synergistic agglomeration. According to the theoretical analysis in this paper, after reaching a certain degree of industrial synergistic agglomeration, it will have a certain negative impact on public health levels. After 2014, the coefficients of industrial synergistic agglomeration and public health levels between the Treat and Control groups showed significant differences. The Treat group showed an upward trend, while the Control group maintained a parallel trend. This result indicates that the DID test conducted in this paper is robust.

## 6 Limitations

This study has several limitations that can be addressed in future research.

First, although city fixed effects and various control variables were incorporated into the model, the heterogeneity across cities (e.g., city size, development level, policy environment) has not been fully considered. Different types of cities may exhibit distinct patterns in the health effects of industrial agglomeration. For instance, mega-cities, due to their stronger environmental regulation and better distribution of health resources, may be more capable of mitigating the adverse health effects of industrial agglomeration, whereas small-and medium-sized cities might face greater health challenges due to limited resources.

Second, the selection and application of instrumental variables are critical for the GS2SLS method. This study relies on spatial distance and characteristics of neighboring cities as instrumental variables but lacks sufficient theoretical justification to establish the exogeneity and exclusivity of these instruments (i.e., that the instruments are correlated with the dependent variable only through the explanatory variables and not through other channels). This raises concerns about the potential invalidity of the instruments, which may undermine the robustness of the estimation results.

Third, this study uses geographic distance and economic distance weight matrices to capture spatial effects. However, spatial dependence in economic research is often influenced not only by geographical proximity but also by various factors such as socio-economic linkages, trade flows, and technology spillovers. Using a single form of spatial weight matrix may lead to underestimation or misestimation of the spatial effects. Future studies could benefit from exploring alternative or multiple forms of spatial weight matrices to better capture the complexity of spatial interactions.

## 7 Conclusions and policy implications

Using a sample of 283 prefecture-level cities in China from 2003 to 2020, this study systematically examines the impact and mechanisms of industrial synergy agglomeration on urban public health by employing the Generalized Spatial Two-Stage Least Squares (GS2SLS) method. In particular, the research explores the heterogeneous effects of green industrial agglomeration and traditional industrial agglomeration on urban public health. The main findings are as follows:

1. Positive Effects of Green Industrial Synergy on Public Health
  The results indicate that, compared to traditional industrial agglomeration, green industrial synergy has a significant positive impact on urban public health. Specifically, the empirical analysis shows that green industry agglomeration reduces the overall incidence and mortality rates and improves residents’ health status. The underlying mechanism lies in the reduction of environmental pollution and enhancement of environmental quality, which contribute to better health outcomes for residents. Additionally, green agglomeration promotes environmental awareness and the formation of a health-conscious culture in urban areas. These effects can generate a “green spillover effect” over a wider geographical area, thereby improving public health in neighboring regions.
2. Significance of Spatial Spillover Effects
  The findings reveal that the effect of industrial synergy agglomeration extends beyond local public health, exhibiting significant spatial spillover effects. This suggests that the agglomeration effect in one city not only influences local public health but also affects the health status of neighboring cities through cross-border pollution and economic linkages. The spatial spillover effects operate through two main channels: first, cross-border pollution (e.g., trans-regional diffusion of air pollutants) negatively impacts the health of residents in adjacent areas; second, economic linkages and resource flows (e.g., the movement of health resources and medical facilities) may exert positive health spillover effects on neighboring regions.
3. Mediating Mechanisms of Population Agglomeration, Media Dissemination, and Income Growth
  The empirical results indicate that population agglomeration, media dissemination, and income growth serve as three key mediating mechanisms through which green industrial synergy influences public health.

First, Population Agglomeration Mechanism: The agglomeration effect of green industries attracts a more educated and health-conscious labor force, fostering the development of a health-oriented culture and healthy behaviors within cities.

Second, Media Dissemination Mechanism: The development of green industries often garners higher public attention and media coverage, thereby enhancing public awareness of environmental protection and health knowledge, which in turn encourages residents to adopt healthier lifestyles.

Third, Income Growth Mechanism: Green industrial agglomeration generates higher economic returns and income levels, enabling residents to invest more in health-related consumption (e.g., healthy food and fitness activities) and healthcare services.

Based on the above mechanisms, the following policy recommendations are proposed:

1. Promote Green Industrial Agglomeration to Improve Public Health
  Governments should increase policy support for green industries (e.g., renewable energy, environmental technology, and energy-saving services) and guide industrial agglomeration through fiscal incentives and green financing. At the same time, stringent environmental standards and regulations should be established to prevent long-term negative health effects resulting from the excessive concentration of traditional industries.
2. Optimize Urban Planning to Enhance Equitable Distribution of Health Resources
  When formulating urban development plans, policymakers should consider adjustments to industrial structure and the spatial distribution of health resources. In regions with industrial agglomeration, governments should develop supporting healthcare facilities and promote healthy behaviors and lifestyles. For highly industrialized cities, particular attention should be paid to pollution control and health risk prevention, especially in providing health support to vulnerable low-income groups.
3. Strengthen Regional Collaboration and Establish Cross-Regional Health Governance Systems
  Given the spatial spillover nature of health effects, public health governance requires coordination and cooperation at the regional level. Governments should establish cross-regional health governance platforms to share health data and medical resources and jointly implement cross-border pollution control measures.
4. Enhance Public Environmental and Health Awareness
  Governments and media should actively engage in health education and environmental protection campaigns to raise public awareness of health issues and environmental responsibility. Through social participation and public oversight, green transformation of the industrial structure can be effectively promoted, thereby improving overall public health levels.

These recommendations aim to provide actionable strategies for optimizing the health benefits of green industrial agglomeration and addressing the complex spatial dynamics in urban public health.

## Data Availability

The original contributions presented in the study are included in the article/supplementary material, further inquiries can be directed to the corresponding author.
